# Dupuytren’s Disease Extending into the Volar Pulp: A Case Report

**DOI:** 10.3390/reports9020139

**Published:** 2026-04-29

**Authors:** Ishith Seth, Sai-Vignesh Ashok, Omar Shadid, Warren Rozen, Snehal Shah

**Affiliations:** 1 Surgery, Peninsula Health, Melbourne 3199, Australia; 2 Plastics and Reconstructive Surgery, Peninsula Health, Melbourne 3199, Australia; 3 Surgery, Monash University, Melbourne 3800, Australia; 4 Plastics and Reconstructive Surgery, Austin Hospital, Melbourne 3084, Australia

**Keywords:** digital deformity, distal interphalangeal joint, Dupuytren’s disease, limited fasciectomy, volar pulp

## Abstract

**Background and Clinical Significance:** Dupuytren’s disease (DD) typically affects the palmar fascia and proximal digital structures, with distal interphalangeal joint (DIPJ) involvement considered rare. True extension of DD into the volar pulp has not been previously documented. Distal lesions may be misdiagnosed as neoplastic or inflammatory masses, and optimal management of isolated distal cords remains uncertain. We present the first histologically confirmed case of DD extending beyond the DIPJ into the volar pulp, accompanied by a systematic review of reported DIPJ-dominant DD. **Case Presentation:** A 30-year-old right-hand-dominant male presented with a two-year history of progressive flexion deformity of the little finger. Examination demonstrated a 90° proximal interphalangeal joint and 55° DIPJ contracture. Ultrasound and MRI showed a well-circumscribed soft-tissue lesion along the radial middle phalanx but did not suggest DD. Open exploration via an ulnar digital approach revealed a discrete DD cord extending distally beyond the DIPJ into the volar pulp, closely associated with the ulnar neurovascular bundle. Limited fasciectomy achieved full correction without neurovascular compromise. Histopathology confirmed classic DD. At the twelve-month follow-up, the patient maintained full extension and function with no recurrence. **Conclusions:** This study reports the first confirmed case of DD extending into the volar pulp and highlights that atypical distal DD can occur even in young patients. Imaging may fail to identify DD in uncommon sites, reinforcing the importance of clinical suspicion. Limited fasciectomy remains safe and effective in the distal phalanx. Recognition of this phenotype or histopathological examination may improve diagnostic accuracy and guide tailored operative planning.

## 1. Introduction and Clinical Significance

Dupuytren’s disease (DD) is a benign fibroproliferative disorder of the palmar and digital fascia, characterised by abnormal collagen deposition, myofibroblast proliferation, and the formation of abnormal cords [1,2]. These cords cause progressive flexion contractures of the fingers, most often involving the metacarpophalangeal (MCP) and proximal interphalangeal (PIP) joints [3,4]. The pretendinous and central cords are usually involved in these deformities, contributing to the typical clinical pattern seen in early-stage disease [5].

In contrast, contractures involving the distal interphalangeal (DIP) joint are uncommon. This is due to anatomical factors, as DIP joint involvement usually requires the disease to extend into the lateral digital or retrovascular cords, structures that are less frequently affected in DD [6,7]. These cords are localised and situated deep within the finger and near neurovascular bundles, making their involvement less common and management more technically challenging [6]. Consequently, isolated DIP joint contractures are rarely reported in the literature and are considered an unusual manifestation of the condition.

The first documented cases of isolated DIP disease were reported by Bellonias and Nancarrow in 1991, who described two middle-aged men with cords affecting the DIP joint of the little finger, both following trauma [6]. The pathological cords extended to the dorsum of the distal phalanx and nail bed, a pattern not previously recorded. Since then, several other case reports have described similar findings [8,9]. Rao et al. reported an isolated DIP contracture in the small finger [8], and Saleh et al. documented Dupuytren’s disease confined to the interphalangeal joints, with cords on both radial and ulnar aspects of the little finger [9]. Most of these patients were male and over 60 years of age, and the majority of affected digits were little fingers with radial cord involvement.

Despite these reports, distal disease remains poorly characterised. Combined isolated contractures of the PIP and DIP joints without proximal palmar involvement are rarely described, particularly in younger patients and in cases extending beyond the DIP joint into the fingertip pulp, the latter of which, to our knowledge, has not been previously reported.

In this context, we present the case of a 30-year-old male with Dupuytren’s disease extending from the PIP joint to the fingertip pulp, resulting in an isolated PIPJ and DIPJ flexion contracture. This case is particularly unusual due to the patient’s young age, the absence of preceding trauma or systemic risk factors, and the distal extent of fascial involvement extending beyond the DIP joint to the volar pulp. It highlights the importance of recognising atypical disease patterns and the surgical considerations involved in treating isolated digital contractures.

## 2. Case Presentation

A 30-year-old right-hand-dominant male presented with a two-year history of progressive flexion contracture affecting the PIPJ and DIPJ of the right little finger. The condition began sporadically at the PIPJ and gradually progressed to involve the DIPJ. He had not received any prior treatment for this condition and reported increasing difficulty with hand function, particularly during fine motor tasks such as playing musical instruments.

There was no family history of DD, and no evidence of Dupuytren’s diathesis was found. The patient had a medical history of autism spectrum disorder, for which he was unmedicated. He was otherwise healthy, with no regular medications and no known drug allergies.

**Figure 1:**
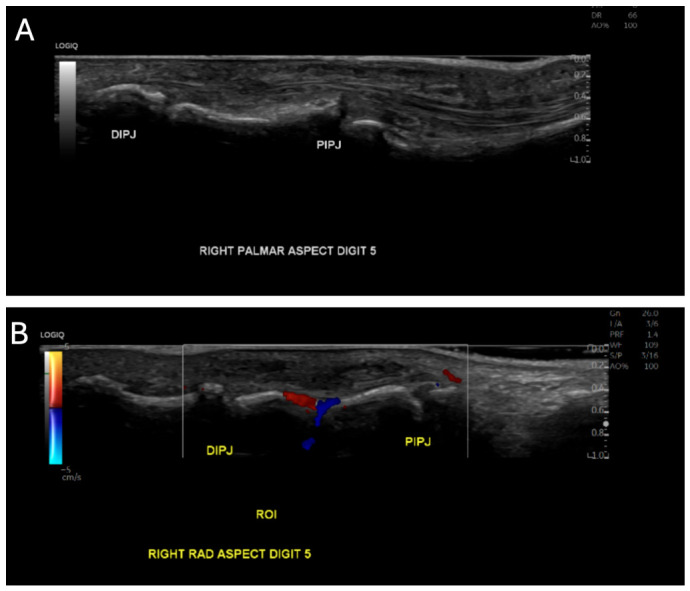
Initial ultrasound imaging of the right little finger. (**A**) Palmar aspect ultrasound demonstrating the lesion within the right little finger. (**B**) Radial aspect ultrasound of the right little finger demonstrating the lesion from a radial approach.

Physical examination revealed a fixed flexion deformity of approximately 90° at the PIPJ and 55° at the DIPJ. Initial high-resolution ultrasound (US) performed in early 2024 demonstrated mild subcutaneous soft tissue thickening over the radial aspect of the little finger PIPJ (Figure 1A,B).

**Figure 2:**
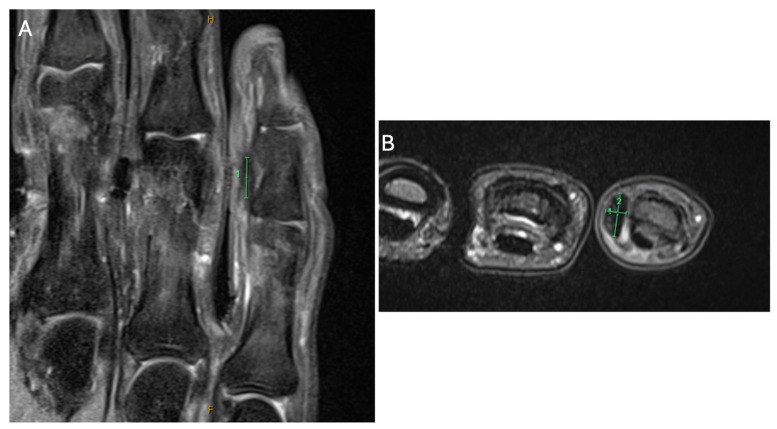
Magnetic resonance imaging (MRI) of the right little finger. (**A**) Coronal MRI demonstrating the longitudinal extent of the lesion along the digit. (**B**) Axial MRI section of the right little finger demonstrating the lesion in cross-section.

Importantly, the flexor and extensor tendons were intact and uninvolved, with no abnormal vascularity or foreign body identified. The radiologist’s interpretation was non-specific, with a differential diagnosis including soft tissue inflammation or a partially ruptured ganglion, and a recommendation for magnetic resonance imaging (MRI) if the lesion persisted (Figure 2). Subsequent MRI confirmed the presence of a 7 mm T1- and T2-hypointense lesion within the subcutaneous tissues overlying the radial aspect of the middle phalanx. The lesion demonstrated minimal heterogeneous enhancement and was associated with surrounding subcutaneous oedema (Figure 2).

**Figure 3:**
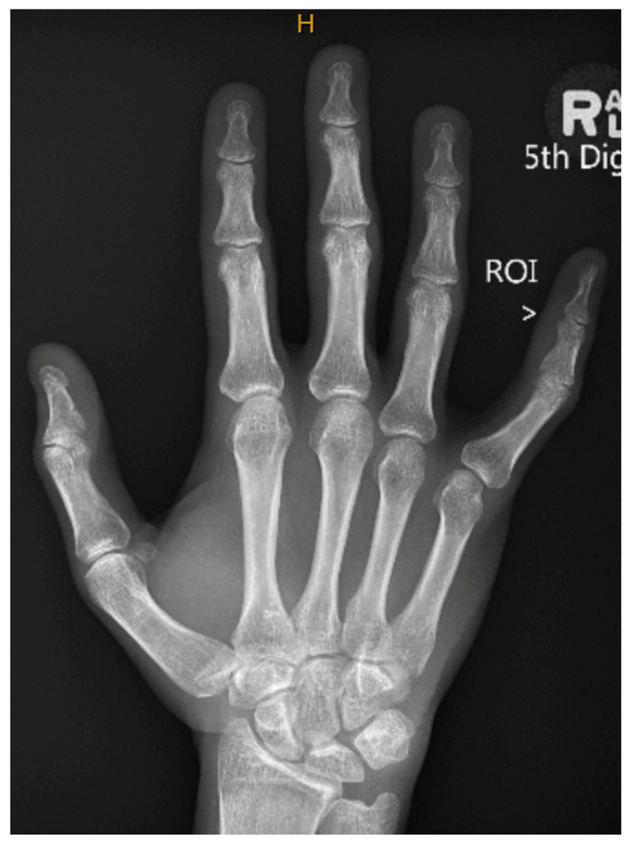
Plain Radiography of the Right Little Finger.

The lesion showed no clear communication with the flexor tendon sheath or extensor mechanism, and no joint or bone abnormalities were identified. The radiological differential diagnoses included a giant cell tumour of the tendon sheath, fibroma, or fasciitis, with features atypical for haemangioma or peripheral nerve sheath tumour. Notably, DD was not suggested in the imaging reports, reflecting its atypical distal presentation and anatomical location beyond commonly affected fascial planes. The x-ray was radiologically unremarkable from a skeletal standpoint but supported the clinical impression of soft tissue pathology isolated to the volar aspect of the little finger (Figure 3).

**Figure 4:**
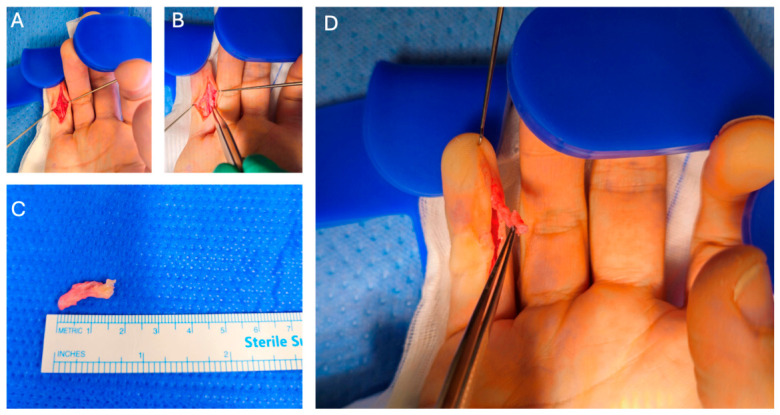
Intra-operative photographs of a distal Dupuytren’s cord. (**A**) Volar approach to the right little finger demonstrating exposure of the distal cord following skin incision. (**B**) Careful dissection and isolation of the distal Dupuytren’s cord from surrounding soft tissues. (**C**) In situ appearance of the distal cord prior to excision, highlighting its relationship to the digital structures. (**D**) Excised distal Dupuytren’s cord specimen measured on a sterile ruler.

Despite inconclusive imaging, the clinical suspicion of Dupuytren’s disease remained high based on progressive flexion contracture and the absence of trauma, neoplastic history, or inflammatory markers. The patient proceeded to surgery. Surgical exploration was performed via a longitudinal incision over the ulnar aspect of the right little finger. Intra-operatively, a well-defined pathological Dupuytren’s cord was identified, originating at the PIPJ and extending distally to the volar pulp of the distal phalanx, a location not previously described in the literature (Figure 4). The cord was dissected and meticulously excised, with particular care taken to protect the adjacent ulnar neurovascular bundle, which was intimately associated with the diseased fascia in the distal phalanx (Figure 4). The wound was closed using interrupted 5-0 chromic gut sutures and dressed with simple dressings and volar extension plaster of Paris.

**Figure 5:**
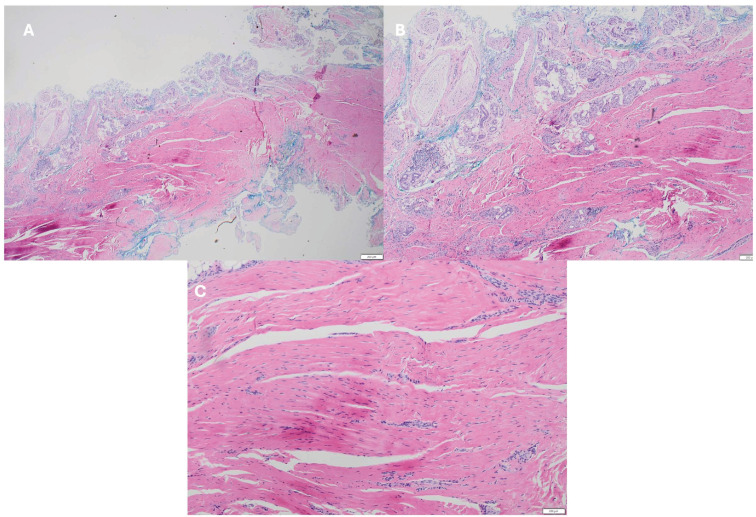
Histopathological features of the excised distal Dupuytren’s cord. (**A**) Low-power view (×20) demonstrating dense, disorganized collagen bundles within the fibrous cord, with involvement of adjacent soft tissues. (**B**) Intermediate-power view (×40) showing thickened collagen fibres with interspersed fibroblasts and myofibroblasts, consistent with fibroproliferative disease. (**C**) High-power view (×100) highlighting tightly packed collagen bundles and elongated spindle-shaped fibroblasts without features of malignancy.

The excised tissue was sent for histopathological analysis, which confirmed the diagnosis of Dupuytren’s disease, demonstrating classic features of DD of variable cellular proliferation of uniform, spindled fibroblasts arranged in nodules composed of long sweeping fascicles within collagenous stroma (Figure 5).

**Figure 6:**
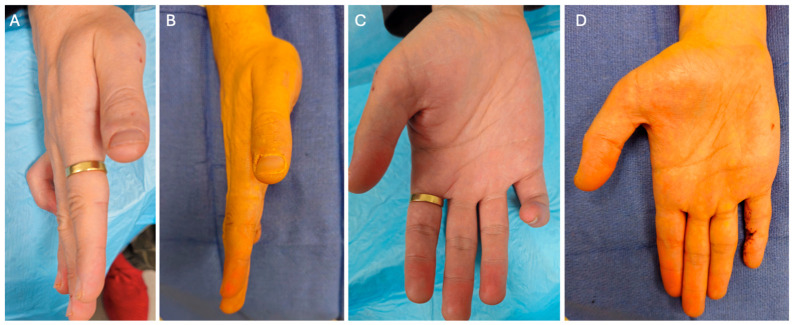
Pre- and post-operative clinical outcome following excision of a distal Dupuytren’s cord. (**A**) Pre-operative lateral view demonstrating distal interphalangeal joint flexion contracture of the right little finger. (**B**) Post-operative lateral view demonstrating correction of the distal interphalangeal joint flexion contracture. (**C**) Pre-operative volar view showing flexion deformity of the right little finger. (**D**) Post-operative volar view demonstrating improved finger extension and hand posture.

Post-operatively, the patient achieved full passive extension, with correction from 90° to 0° at the PIPJ and from 55° to 0° at the DIPJ (Figure 6). He was fitted with a custom night extension splint for six weeks and commenced supervised hand therapy to optimise the range of motion and function.

**Figure 7:**
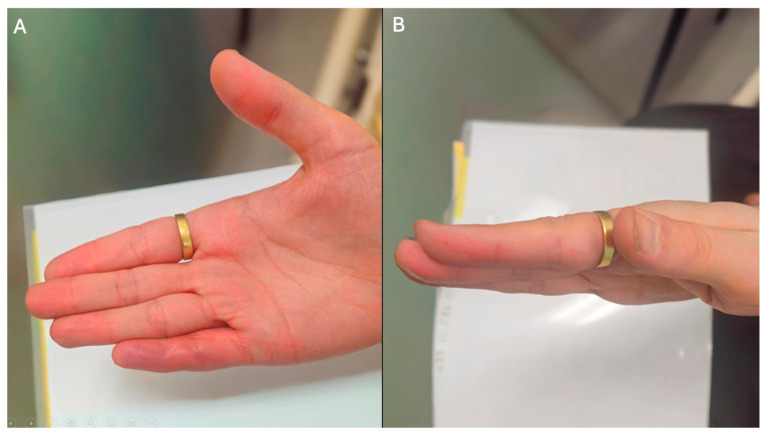
Twelve-month post-operative clinical outcome following excision of a distal Dupuytren’s cord. (**A**) Volar view demonstrating sustained correction of the distal interphalangeal joint contracture at 12-month follow-up. (**B**) Lateral view showing maintained finger extension and functional alignment at 12 months post-operatively.

At the twelve-month follow-up, the patient reported significant improvement in hand function with no evidence of recurrence or complications (Figure 7). There was 0° contracture at both the PIPJ and DIPJ, with full active extension maintained at both joints. The patient resumed playing musical instruments and demonstrated no sensory or motor deficits. This case highlights the importance of correlating imaging with clinical findings in atypical digital presentations and supports early surgical intervention in isolated digital Dupuytren’s disease with distal extension.

## 3. Discussion

Isolated involvement of DD into the distal interphalangeal joint is rare, reported in fewer than 5% of all cases, and disease extending beyond the DIPJ to the volar pulp has not been previously described in the published literature [7,10,11]. To our knowledge, this is the first documented case of Dupuytren’s disease with histologically confirmed extension to the distal third of the volar pulp in an otherwise healthy young adult without systemic risk factors, Dupuytren’s diathesis, or family history. This report not only highlights a highly atypical anatomical localisation but also contributes novel insights into the distal extent of fascial involvement in Dupuytren’s disease.

Anatomically, the presence of Dupuytren’s disease in the volar pulp is highly unusual and has not been previously explained in the literature. This rarity is likely due to the absence of well-defined fibroaponeurotic structures in the distal third of the digit [12]. Dupuytren’s disease typically affects the pretendinous band, spiral band, central cord, lateral digital sheet, Grayson’s ligament, and retrovascular cords—structures that are primarily located proximal to the distal interphalangeal joint [2]. Notably, Grayson’s ligaments, which are implicated in digital contracture formation, terminate before reaching the distal phalanx and do not extend into the pulp [12]. The volar pulp is composed mainly of adipose tissue, neurovascular structures, and loosely arranged fibrous septae, which lack direct anatomical continuity with the diseased cords seen in more proximal disease [13].

We hypothesise that, in this case, disease progression beyond the distal interphalangeal joint into the pulp may reflect either an aggressive or aberrant fascial phenotype, possibly involving atypical fibrous bands or retrovascular extensions not typically seen in classical Dupuytren’s disease. Alternatively, it may represent a rare variant of digital involvement in which myofibroblast-driven proliferation occurs within the distal septal system independent of the traditional cord network. This case provides the first clinical and histological evidence that Dupuytren’s disease can, albeit rarely, extend beyond recognised anatomical boundaries into the fingertip pulp.

From a surgical standpoint, managing distal digital disease poses unique challenges. The neurovascular bundles in the distal phalanx run near the skin and are more vulnerable to iatrogenic injury, particularly when the disease extends near the fingertip [13]. A significant complication of the surgical treatment of DD is neurovascular damage; for this reason, the dissection and removal of the chord should be done under loupe magnification, regardless of whether it is a complete or partial fasciotomy. The operation achieved complete correction of deformity at both the PIPJ and DIPJ, with histopathological confirmation of Dupuytren’s disease in the excised cord. The successful outcome without complications or recurrence at six months further supports the safety and efficacy of limited fasciectomy in well-selected distal cases, even those involving the pulp.

Reviewing the literature, only nine studies have previously documented isolated digital involvement at the DIPJ, and none included extension to the pulp. Of these, the majority were treated with surgical fasciectomy, while only two cases utilised CCH injection. Mehdi et al. (2019) and Stanley & Cavallo (2020) demonstrated that CCH could yield meaningful correction at the DIPJ, but its safety profile in such distal and anatomically complex zones remains unclear [11,14]. Concerns persist regarding the proximity of pathological cords to the neurovascular bundles, which may be inadvertently affected during enzymatic degradation [15]. Given these anatomical constraints, our case supports the preferential use of surgical intervention in anatomically distal or atypically located cords.

Another critical consideration is recurrence. In general, recurrence following treatment of Dupuytren’s disease, particularly in the small finger and in younger patients, is a common occurrence [15]. However, our patient exhibited no recurrence at six-month follow-up, which may be attributable to the completeness of fascial excision and the absence of other diathesis features. While long-term surveillance is warranted, this early outcome is encouraging.

This case also prompts broader reflection on how atypical Dupuytren’s disease may present in younger patients and be overlooked or misdiagnosed due to its divergence from classical teaching. Current staging systems, including the Tubiana classification, quantify disease severity based on the degree of digital flexion contracture. However, these systems and associated clinical guidelines were developed largely from cohorts with typical palmar Dupuytren’s disease in older males, which may limit their applicability to atypical presentations such as isolated distal digital involvement [16]. As such, presentations involving distal anatomy, isolated digital cords, or early-onset manifestations in patients without identifiable risk factors may fall outside the threshold of clinical suspicion, particularly among non-hand-specialist practitioners. It is conceivable that such cases are underreported or misattributed to benign soft tissue lesions, tendon pathology, or post- traumatic scarring. This raises the possibility of a distinct, under-recognised end of the Dupuytren’s disease spectrum, one that is not necessarily driven by genetic diathesis or systemic fibrotic tendencies but may reflect sporadic or epigenetic alterations in fascial fibroblast behaviour [17]. Future work exploring the molecular phenotype of isolated digital cords in young patients may reveal important insights into alternative pathomechanisms of disease. Until then, this case underscores the importance of anatomical vigilance and a low diagnostic threshold in the presence of unexplained progressive digital deformity, particularly when imaging is inconclusive.

While Dupuytren’s disease is primarily a clinical diagnosis and does not typically require imaging for confirmation, the use of imaging adjuncts such as ultrasound or MRI may be appropriate in selected scenarios. In this case, given the patient’s unusually young age, the absence of Dupuytren’s diathesis, and the highly distal location of the pathology, it was important to exclude sinister differential diagnoses such as soft-tissue neoplasms or inflammatory masses. Both ultrasound and MRI were performed but yielded non-specific findings, highlighting the potential for imaging to mischaracterise distal Dupuytren’s pathology when it masquerades as other lesions. In situations where imaging findings are incongruent with intraoperative appearances, histopathological examination of the excised tissue remains essential to confirm the diagnosis and exclude alternative pathology. This underscores the continued importance of clinical judgement in evaluating atypical digital deformities, particularly in the context of ambiguous imaging.

This study has several limitations. First, as a single case report, the findings cannot be generalised to the broader population of patients with Dupuytren’s disease. The unusual age of onset and atypical anatomical distribution limit external validity, and longer follow-up is required to confirm durability of correction and recurrence risk. Second, the systematic review is based exclusively on published case reports and small case series, which are inherently subject to publication bias, incomplete reporting, and heterogeneity in outcome measurement. Given the rarity of DIPJ involvement, many cases may remain unpublished or under-recognised, leading to underestimation of prevalence and disease spectrum. Third, although a structured screening and data extraction process with independent reviewers was employed, formal quantitative synthesis was not feasible due to the small number of cases and reporting heterogeneity, limiting comparative conclusions regarding management strategies or long-term outcomes.

## 4. Conclusions

This case represents the first histologically confirmed instance of Dupuytren’s disease extending beyond the distal interphalangeal joint to involve the volar pulp, and this case is the youngest reported patient to exhibit such distal digital involvement. In the absence of known risk factors, diathesis, or familial history, this presentation challenges existing paradigms and highlights a previously undocumented anatomical frontier of the disease. It underscores the need for heightened clinical suspicion when assessing atypical digital contractures, particularly in younger patients. When imaging findings are inconclusive or non-specific, histopathological examination remains important for confirming the diagnosis and excluding alternative pathology. Beyond its rarity, this case broadens the recognised phenotypic spectrum of Dupuytren’s disease and reinforces the value of individualised, anatomy-informed surgical planning to achieve safe and effective outcomes in complex digital pathology.

## Data Availability

The original contributions presented in this study are included in the article. Further inquiries can be directed to the corresponding author.
